# How do hospitals exert market power? Evidence from health systems and commercial health plan prices

**DOI:** 10.1093/haschl/qxae179

**Published:** 2025-01-16

**Authors:** Suhui (Evelyn) Li, David Jones, Eugene Rich, Aimee Lansdale

**Affiliations:** Mathematica, Princeton, NJ 08540, United States; Mathematica, Princeton, NJ 08540, United States; Mathematica, Princeton, NJ 08540, United States; Mathematica, Princeton, NJ 08540, United States; University of California, Department of Epidemiology and Biostatistics, San Francisco, CA 94158, United States

**Keywords:** health systems, hospitals, commercial insurers, market concentration, negotiated prices

## Abstract

Consolidation of independent hospitals and physician practices into integrated health systems has reshaped the delivery of health care. While the literature suggests that provider consolidation raises prices, few studies have examined the interplay of health systems and insurers in relation to prices. Using negotiated price data that commercial insurers recently released under the Transparency in Coverage Final Rule, we examined the association between hospital concentration under health systems and prices for outpatient procedures in local health care markets with different levels of insurer concentration. We found that hospital prices are higher in more concentrated hospital markets, while lower in more concentrated insurer markets. However, the negative relationship between insurer concentration and hospital prices is attenuated in highly concentrated hospital markets, suggesting that insurers' bargaining leverage is lessened at greater levels of hospital consolidation. Considering the continued consolidation among hospitals and vertical integration of physician practices into health systems, our findings suggest that commercial payers may encounter increased challenges in controlling health care spending for their beneficiaries as providers' bargaining power continues to grow.

## Introduction

In the past several decades, health systems have reshaped the local health care landscapes across the country by acquiring not just local hospitals but also independent physician practices to form vertically integrated organizations delivering comprehensive services.^1^ As of 2021, these health systems exert control over many key health care providers, with 93% of acute care hospital beds and 52% of physicians falling under their purview.^2^ While health system advocates have argued that integration can yield operational efficiencies and quality-of-care enhancements, research suggests that hospital consolidation leads to increased costs and prices without a corresponding improvement in care quality.^3,4^ Furthermore, integration of physicians with hospitals has been associated with increased payments by both Medicare and commercial insurers, in part from the redirection of services from office settings to higher-cost outpatient departments.^5-8^

Private insurance plans have also become more concentrated in many local markets.^9^ Economic theory predicts that concentration of private insurers gives them the bargaining power to negotiate lower prices with providers. Melnick et al^10^ found that hospital prices in the most concentrated health plan markets are lower than in more competitive markets. More recently, Roberts et al^11^ and Wang et al^12^ found that major insurers pay lower negotiated rates to physician groups and hospitals for shoppable outpatient services. As both markets become more consolidated, however, how insurer concentration intertwines with provider concentration to influence prices is not well understood.

The objective of this study was to examine how the concentration of hospitals under health systems and private insurers in local health care markets correlate with facility prices at hospital outpatient departments (HOPDs). Compared with inpatient procedures for which price competition is often softened by specialization and quality differentiation among local hospitals, common outpatient procedures make up a more crowded market as patients have more options among local hospitals and surgical centers that provide those procedures.

Today, most local markets are highly penetrated with health systems, which negotiate prices with insurers on behalf of all their hospitals.^13^ Therefore, it is important to consider the role of health systems in measuring hospitals' bargaining power. We define a hospital-system entity as a group of hospitals within a market associated with a health system or a single independent hospital.

We hypothesized that hospital prices are higher in markets with a higher concentration of hospital systems and lower in markets with a higher concentration of insurers. However, the net effect of the hospital–insurer dynamic is an empirical question. It is possible that concentration of insurers creates a greater incentive for hospitals to compete on prices, allowing insurers to extract rents from their bargaining power. In such a case, we would expect to see a negative relationship between insurer concentration and prices when there is hospital competition. But, as the hospital market becomes more concentrated and closer to a monopoly, we might see a weak relationship between insurer concentration and prices because insurers are less effective in negotiating lower prices in such markets.

Our study contributes to the literature in several important ways. First, we add new evidence to the study of how the bargaining power of health care providers and insurers affects prices. Second, in most of the literature, market concentration of hospitals is measured based on market shares of individual hospitals, which ignores the influence of health systems and thus underestimates the true level of concentration. In this paper, we address this issue by identifying hospital affiliation with health systems and calculating market concentration based on market shares of hospital-system entities. Last, while prior studies examined average hospital prices by using commercial claims data in the early 2010s, we examined the actual contracted rates by using the payer price transparency data that health plans published under the Transparency in Coverage (TiC) mandate of 2022.^14^ Evidence drawn from this novel dataset contributes to the existing literature built mainly on claims data.

## Data and methods

### Price data and study sample

We used the commercial contracted rates data published by commercial health plans under the 2022 TiC Final Rule. Under the rule, most commercial health plans in individual and group markets, including employer-sponsored plans, collectively released massive volumes of rates data in hundreds of thousands of machine-readable files (MRFs). To manage the data size for this study, we focused on United Healthcare (UHC) and Aetna—2 large insurers that have market shares in most health care markets across the nation. We downloaded the payers' raw MRFs in November 2023 and extracted raw rate files for Preferred Provider Organization (PPO) plans—the most common type of plan among the working population with employer coverage.^15^ We then focused on analyzing pricing data for outpatient procedures commonly performed in hospitals. We did not have data on service utilization for the commercially insured population to identify services with the highest volumes, so we relied on a list of services that California, the state with the largest population, considers as common outpatient procedures. Specifically, the California Department of Health Care Access and Information requests hospitals to report annual outpatient charges on a list of surgery, medicine, and other services.^16^ We used the 17 surgery services from the list, including knee arthroscopy, carpal tunnel surgery, cataract procedures, colonoscopy, endoscopy, hernia repair, epidural steroid injection, laparoscopy, tympanotomy, and tonsillectomy (the list of Current Procedural Terminology [CPT] codes is included in Appendix SII, Figure S1). Based on the authors' analysis of the Medicare fee-for-service (FFS) claims, these CPT codes were associated with approximately 2 million Medicare outpatient claims in 2023 and accounted for 3% of Medicare payments for all outpatient services. We acknowledge that these codes represent a small fraction of all outpatient billing codes, and the results may not be representative of other services.

Our study sample includes 42 145 prices for the 17 billing codes from 1932 HOPDs. Details of data-processing steps and billing codes included in the sample are shown in Appendix SI. To account for price variations due to geographic differences, we estimated the Medicare relative rates by taking the ratio between the commercial contracted rate and what Medicare FFS would have paid to the same provider for the same service.

### Measures of hospital-system and insurer concentration

We define health care markets as Metropolitan Statistical Areas (MSAs). To measure the level of hospital concentration while accounting for health systems, we used the 2022 Compendium of US Health Systems Hospital Linkage File,^17^ published by the Agency for Healthcare Research and Quality. The file contains a comprehensive list of hospitals, unique Centers for Medicare and Medicaid Services (CMS) certification number (CCN), and unique health system IDs if they are associated with health systems that include at least 1 hospital and at least 1 group of physicians that provides comprehensive care (including primary and specialty care) who are connected with each other and with the hospital through common ownership or joint management. We obtained hospital patient discharge data from the 2022 Hospital Cost Tool (HCT) published by the National Academy of State Health Policy. The HCT dataset calculates hospital-adjusted patient discharges as the number of inpatient discharges multiplied by the ratio between total hospital charges and inpatient hospital charges. We used this variable to measure hospital-system market shares that account for both inpatient and outpatient volumes.

We then aggregated the hospital data to construct 2 MSA-level measures: (1) “system dominance” calculates the percentage of hospital beds controlled by the largest health system and (2) “hospital-system concentration” calculates Herfindahl Hirschman Index (HHI), which is the sum of the squared market shares (based on adjusted patient discharges) of all health systems and independent hospitals within an MSA. As mentioned above, we defined a hospital-system entity as either a single hospital not associated with any health system or all hospitals associated with the same health system within an MSA. For example, suppose that an MSA has 4 hospitals each with a 25% market share, but 3 of the 4 hospitals were affiliated with 1 health system. Under our method, the market shares for hospital-system entities in the MSA would be 25% for the independent hospital and 75% for the system, yielding an HHI of 6250 (=25^2^ + 75^2^). Our method provides an alternative to the more commonly used HHI measure that sums market shares across individual hospitals, which tends to underestimate hospital market concentration (in this example, the traditional measure would yield an HHI of 2500).

We collected data on insurer concentration from the American Medical Association’s 2023 report, which provides the commercial payer market shares and HHIs in MSAs across the nation in 2022.^9^ We linked the above-mentioned market concentration measures with the facility-level prices by MSA to create the final analysis data.

### Analysis

Our primary outcome measure was the commercial contract rates measured as Medicare relative rates at the hospital-billing code level. We estimated 2 linear regression models to examine how market concentrations are correlated with hospital-negotiated service prices. The first model simply estimates the nonlinear correlation between market concentration and Medicare relative rates by including insurer HHI tertiles and hospital-system HHI tertiles as independent variables. To examine the interdependent effect of the 2 market concentrations, our second model includes the full interaction between insurer HHI tertiles and continuous hospital-system HHI. In both models we controlled for factors that could affect both concentration and prices, thus obscuring the direct relationship between the 2. These factors include payer, hospital disproportionate share hospital status and major teaching hospital status, MSA-level population size and median personal income, and billing code fixed effects. We report the regression-adjusted average Medicare relative rates over subgroups of markets based on insurer HHI tertiles and then by low, medium, and high hospital-system concentration categories. We defined low concentration as an HHI below 2500, medium concentration as an HHI between 2500 and 5000, and high concentration as an HHI above 5000. Notably, our threshold for low concentration is already higher than the Department of Justice’s (DOJ's) threshold of high concentration (HHI >1800).^18^ As shown below, most hospital markets fall into the DOJ's definition of high concentration. Therefore, we used higher thresholds to match more closely to the empirical distribution of HHIs across markets. To test the robustness of our results, we performed sensitivity analyses by using alternative regression specifications and sample inclusion criteria.

### Limitations

Several limitations should be considered when examining the results. First, market definition could influence the competitive landscape, particularly in large MSAs (eg, the New York–Newark–Jersey City MSA) where submarkets may exhibit different competitive dynamics. Even though we controlled for MSA sizes in regressions, our results may still be influenced by a few MSAs that are disproportionately larger than others. To address this concern, one of our sensitivity analyses excludes hospitals in the largest MSAs. Second, our analysis does not fully account for the complexity of hospital pricing decisions and various factors influencing the ultimate prices. For example, the literature suggests that there may be cross-service pricing effects where hospitals serving as trauma centers may charge higher prices for non-trauma inpatient and outpatient services.^19^ Also unobserved in-market relationships across ambulatory surgery centers (ASCs) and health systems may affect service prices. For example, competitive pressures from independent ASCs may lower hospital pricing on outpatient procedures,^20^ while unobserved acquisitions of ASCs by health systems may increase prices.^21^ Third, although our price data include 2 national insurers, their negotiated prices with hospitals may not be representative of other payers' negotiated prices, particularly in health care markets dominated by regional and local payers. In addition, we do not have data on the market shares of the 2 payers in individual markets, which may also influence their prices. Fourth, the limited set of outpatient services we examined may not be representative of pricing patterns on other health care services, thus limiting the generalizability of our results. We also do not have data on the service volumes among commercially insured populations, so we could not estimate the volume-weighted correlations of HHIs and service prices. Last, given the descriptive nature of this study, our results should be interpreted as the correlation between market concentration and service prices; the results should not be used to draw definitive causal inferences about the specific changes in prices we should expect with a specific change in market concentration.

## Results

We found that health systems have significant control over hospital beds across MSAs. In more than half of the 376 MSAs, 100% of hospital beds were controlled by health systems in 2022 (Table 1). On average, the largest health system controls 61% of beds in its MSA. The median hospital-system and insurer HHI was 5078 and 3226, respectively. Based on DOJ's HHI threshold of 1800,^18^ most insurer and hospital-system markets are highly concentrated, although insurer markets are, on average, less concentrated than hospital-system markets.

**Table 1.** Health system and insurer concentration across Metropolitan Statistical Areas.

Market characteristics (*n* = 374)	Mean	Minimum	Median	Maximum
Total bed counts	2003	14	597	117 516
Beds associated with any health system	1455	0	565	37 699
Share of beds associated with any system	91%	0%	100%	100%
Share of beds associated with the largest system	61%	0%	58%	100%
Hospital-system HHI	5603	636	5078	10 000
Insurer HHI	3486	1380	3226	8415

Source: Authors' analysis of the 2022 Compendium of US Health Systems Hospital Linkage File published by the Agency for Healthcare Research and Quality and the 2023 report on competition in health insurance published by the American Medical Association. Hospital-system HHIs are calculated based on entities' market shares of adjusted patient discharges, sourced from the 2022 Hospital Cost Tool published by the National Academy of State Health Policy.

Abbreviation: HHI, Herfindahl Hirschman Index.

**Figure 1.**
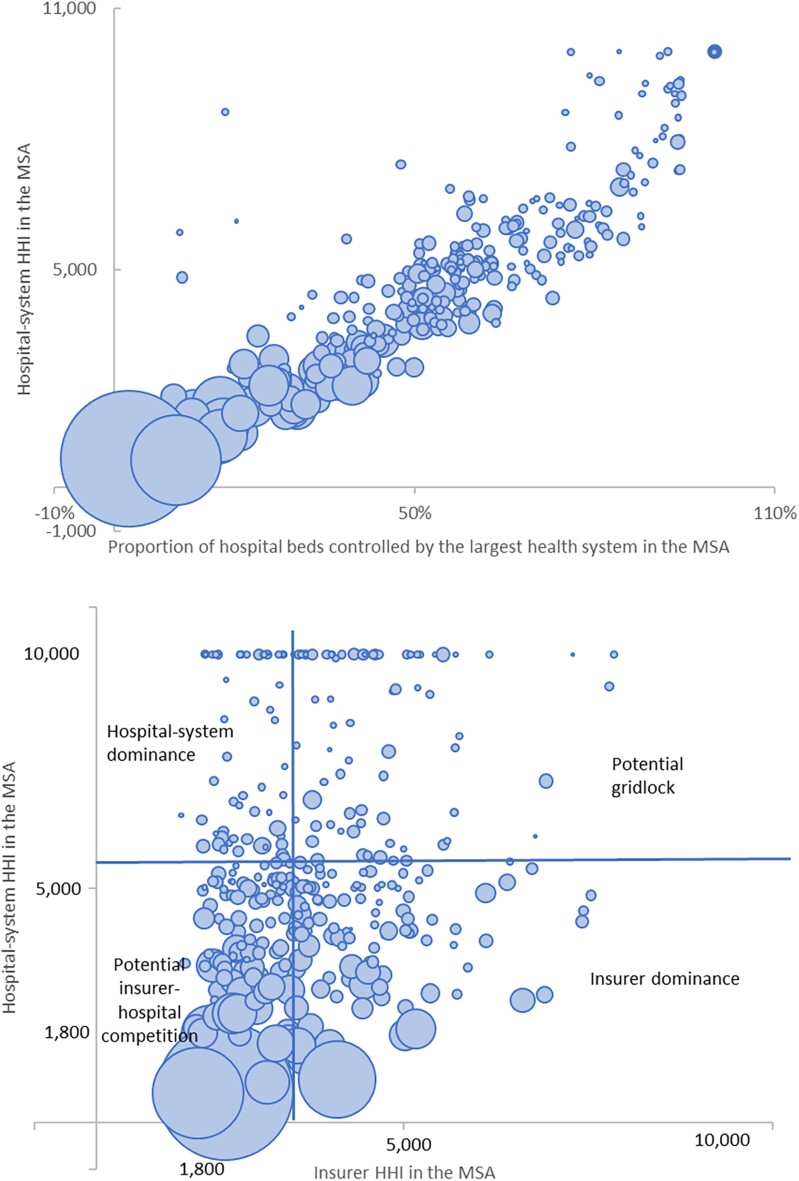
Correlation between hospital-system HHI vs health system dominance and insurer HHI. Source: Authors' analysis of the 2022 Compendium of US Health Systems Hospital Linkage File published by the Agency for Healthcare Research and Quality and the 2023 report on competition in health insurance published by the American Medical Association. Hospital-system HHIs are calculated based on entities' market shares of adjusted patient discharges, sourced from the 2022 Hospital Cost Tool published by the National Academy of State Health Policy. The sizes of the bubbles represent the sizes of the MSAs based on the total number of hospital beds. The gridlines in the lower panel represent the medians of hospital-system HHIs and insurer HHIs. Abbreviations: HHI, Herfindahl Hirschman Index; MSA, Metropolitan Statistical Area.

As expected, the levels of health system dominance and hospital-system HHI are strongly related, with a correlation of 0.92 (Figure 1, upper panel). Visually, most MSAs cluster along the diagonal line, but 4 small MSAs on the upper left corner of the plot deviate from the diagonal with low system dominance and high hospital-system HHI. These are markets where most hospital beds are controlled by a local independent hospital. One example is Yakima, WA, where an independent hospital (Yakima Valley Memorial Hospital) accounts for 82% of total hospital beds in the market. Focusing on markets where health systems play a substantive role, we excluded these 4 markets from our main analyses and included them in our sensitivity analysis.

The hospital-system and insurer HHIs are weakly correlated with a correlation coefficient of 0.16 (Figure 1, plot 2). Based on the median values of hospital-system HHI and insurer HHI, 99 MSAs are in the zone of potential insurer–hospital competition because both markets are relatively less concentrated, 95 MSAs are in the potential gridlock zone where both markets are most concentrated, 83 are in the hospital-system dominance zone, and 86 are in the insurer dominance zone.

The median Medicare relative rate for the outpatient procedures studied is 245% under UHC and 243% under Aetna (Appendix SII, Table S1). These estimates are generally consistent with results from other recent studies comparing commercial insurers' and Medicare FFS rates for outpatient services, which range from 155% to 293%.^22^ The Medicare relative rates also vary widely across services, with average relative rates ranging from 101% for tonsil removal for children (HCPCS 42820) to 341% for cataract surgery (HCPCS 66821) (Appendix SII, Figure S1).

**Figure 2.**
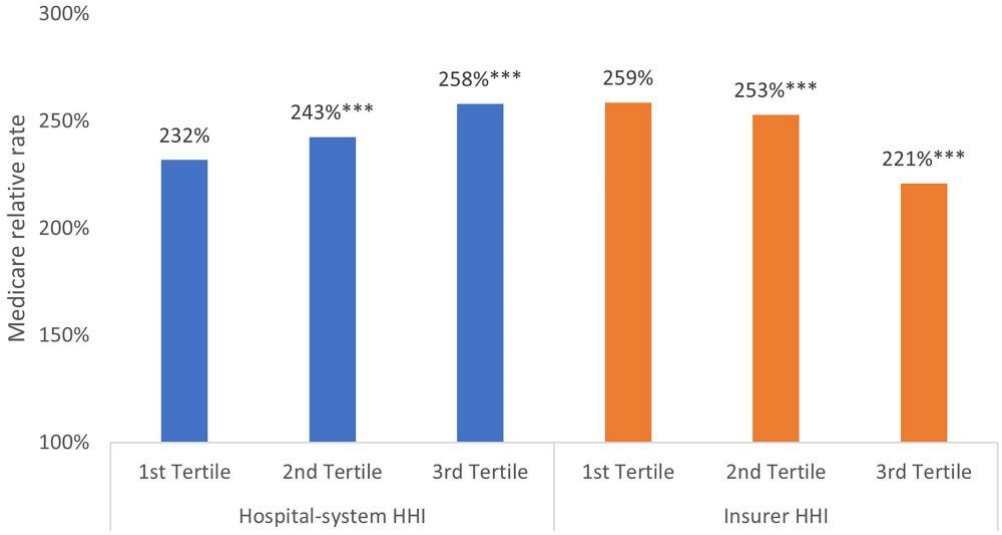
Regression-adjusted average Medicare relative rates by hospitals. Source: Authors' analysis of payer price transparency data published by UnitedHealthcare and Aetna in November 2023, the 2022 Compendium of US Health Systems Hospital Linkage File published by the Agency for Healthcare Research and Quality, and the 2023 report on competition in health insurance published by the American Medical Association. The asterisks denote the level of statistical significance for differences in prices between higher tertiles compared with the lowest tertile of each HHI measure—that is, comparing within the blue bars and orange bars. Regression coefficients are reported in Appendix SII. ***P < .01. Abbreviation: HHI, Herfindahl Hirschman Index.

Figure 2 shows the regression-adjusted Medicare relative rates for hospitals by tertiles of hospital-system and insurer HHIs based on the first regression model. Results show that a greater concentration of hospital systems is associated with higher hospital prices while a greater concentration of insurers is associated with lower hospital prices. Compared with prices in markets with the lowest hospital-system HHIs (232%), prices are 11% and 26% points higher in markets in higher hospital-system HHIs. These differences are statistically significant and represent 5% and 11% higher prices in the second and third HHI tertiles, respectively. Conversely, prices are 6 and 38 percentage points, or 2% and 15%, lower in markets with higher tertiles of insurer HHI than prices in markets with the lowest tertile of insurer HHI (259%).

**Figure 3.**
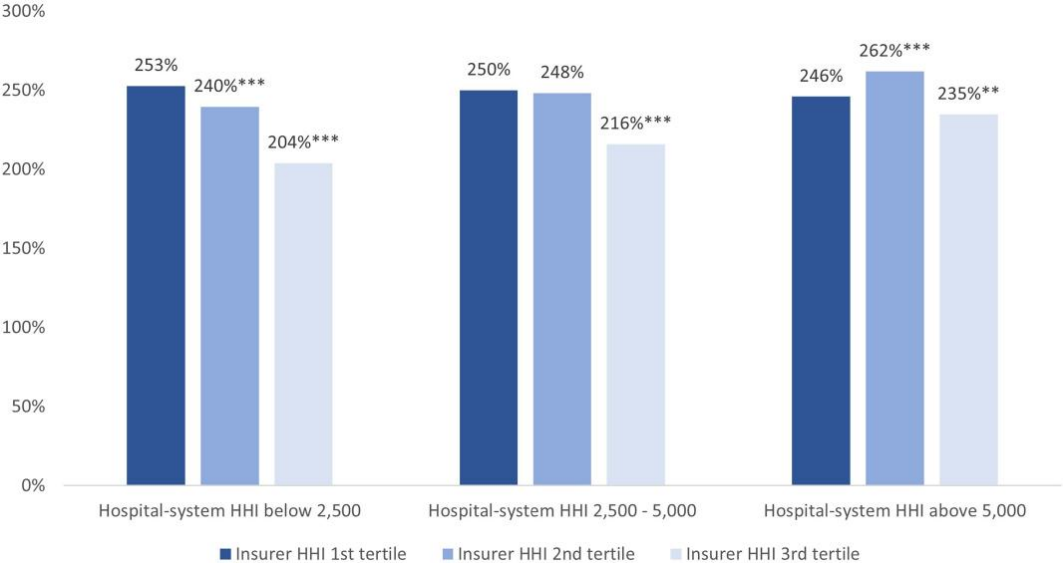
Regression-adjusted average prices for HOPDs by interaction of insurer and hospital-system HHI. Source: Authors' analysis of payer price transparency data published by UnitedHealthcare and Aetna in November 2023, the 2022 Compendium of US Health Systems Hospital Linkage File published by the Agency for Healthcare Research and Quality, and the 2023 report on competition in health insurance published by the American Medical Association. The 3 groups of bars represent average adjusted prices in markets with each group of hospital-system HHIs. Within each group, bars with different shades of blue represent prices in markets with different levels of insurer HHIs. The asterisks denote the level of statistical significance for the price difference between the lowest tertile and higher tertiles of insurer HHIs—that is, comparing the darkest blue with lighter blue bars within a hospital-system HHI category. Regression coefficients are reported in Appendix SII. ***P < .01. Abbreviations: HHI, Herfindahl Hirschman Index; HOPD, hospital outpatient department.

Figure 3 shows the regression-adjusted Medicare relative rates based on linear regressions with full interaction effects between insurer HHI and hospital-system HHI. Results show that, in general, higher insurer HHI is strongly associated with lower hospital prices (ie, the lightest blue bar is lower than the darkest blue bar within each cluster), but the negative correlation between insurer HHI and price is less pronounced with the increase in hospital market concentration. Specifically, when hospital market concentration is below 2500 (the first cluster), the average hospital price in markets with high insurer concentration is 204%, 49 percentage points lower than prices in markets with high insurer concentration. The estimated price differential between low and high insurer concentration shrinks in the second and third clusters. When hospital market concentration is above 5000 (the last cluster), the average hospital price in markets with high insurer concentration is 235%, only 11 percentage points lower than prices in markets with high insurer concentration. Although the pattern is largely consistent across the 3 clusters, it is less clear in the last cluster where hospital prices are the highest in markets with medium insurer concentration.

We performed several sensitivity analyses. First, our hospital-system HHIs identify highly concentrated hospital markets either due to consolidation of local hospitals into health systems or concentration of local independent hospitals. To test the robustness of our results, we ran the analysis using an alternative measure of concentration, the percentage of hospital beds owned by the largest health system in each local market to assess the correlation between health system dominance and prices. Second, to test the influence of large MSAs on our results, we re-estimated our main regressions excluding the 5 largest MSAs, which accounted for approximately 15% of the study sample. Last, because our main regressions excluded 4 MSAs with low system dominance and high hospital-system HHI, we ran a sensitivity analysis including these MSAs. Conclusions from these analyses remain consistent with those from the main analysis. Detailed results are reported in Appendix SII.

## Discussion

In this study, we examined the association between hospital concentration under health systems and prices for outpatient procedures in local health care markets with different levels of insurer concentration. Our results suggest that, overall, concentration of insurers is associated with up to 15% lower hospital prices, while concentration of hospital systems is associated with up to 11% higher hospital prices. However, the negative relationship between insurer concentration and hospital prices is attenuated in highly concentrated hospital markets, suggesting that insurers' bargaining leverage is lessened at greater levels of hospital consolidation.

Our results echo the evidence from Scheffler and Arnold^23^ that insurer market power helps lower provider prices, but we add insights regarding the hospital price implications of interactions between market-level provider and insurer-level concentration. Scheffler and Arnold observed that insurer concentration associated with lower prices in markets with high provider concentration, hypothesizing that, in concentrated provider markets, insurers can potentially extract some of the monopoly rents from providers (whereas there is little rent to extract in low-concentration provider markets because prices would already be close to the competitive level). In contrast, our results suggest that insurer bargaining power decreases when provider market concentration is highest.

The seemingly contradictory findings between our results and Scheffler and Arnold's may be explained by the drastically changing landscapes of provider and insurer markets in the past decade. Notably, Scheffler and Arnold reported a median hospital HHI of 2163 in 2010, while our study found that, in 2022, the median hospital HHI was 5078. Although some of this change in hospital HHIs can be due to differences in data and methods (eg, we accounted for health systems in the HHI measure), it does reflect a substantial increase in the level of hospital concentration documented in the literature.^3^ It is reasonable to believe that the high-concentration hospital markets in Scheffler and Arnold's study (HHIs >2000) largely align with our definition of medium-concentration markets (HHIs between 2500 and 5000). Our findings from medium-concentration hospital markets are therefore consistent with Scheffler and Arnold's finding that, when providers are concentrated, market-dominant insurers can lower prices by extracting rents from providers. In addition, our results also imply that, in extremely concentrated hospital markets (HHIs >5000), insurers may be less effective in exerting their market power against locally dominant health systems. To sum up, the substantial increase in hospital HHIs over the past decade means that many local hospital markets that used to be relatively competitive have become concentrated over time, and some markets that were already concentrated are moving closer to monopoly. As a result, the greater market power gained by health systems could decrease the potential ability of insurers to extract rents and decrease prices.

Finally, although our findings suggest that greater insurer consolidation is associated with lower hospital prices, they do not imply that insurer consolidation is the solution to lowering health care costs. In fact, the literature points to the concern that insurer consolidation can increase health insurance premiums and overall health care spending.^24^

## Conclusion

Our findings underscore the complex dynamics of players in health care markets. In light of the continued consolidation among hospitals and vertical integration of physician practices into health systems, our findings suggest that commercial payers may encounter increased challenges in controlling health care spending for their beneficiaries as providers' bargaining power continues to grow. Therefore, policymakers need to consider both of these market forces in addition to other factors when examining health care delivery markets. In addition, the effects of insurer concentration on overall health care spending, access, and quality of care warrant further investigation. It is important that future research examines the net effect of health system and payer consolidations on the value of care to consumers.

## Supplementary Material

qxae179_Supplementary_Data
