# Supplementary material for: How do hospitals exert market power? Evidence from health systems and commercial health plan prices
Source: Health Aff Sch. 2025 Jan 16;3(1):qxae179. doi: 10.1093/haschl/qxae179 (PMC11736714; doi:10.1093/haschl/qxae179)
Supplement: qxae179_Supplementary_Data [file qxae179_supplementary_data.zip › Appendix_HAS_revised_clean.docx]

# Appendix I. Technical Details on Study Sample

To construct the study sample, we used the commercial contracted rates data published by commercial health plans under the 2022 TiC final rule. Under the rule, most commercial health plans in individual and group markets, including employer-sponsored plans, collectively released massive volumes of rates data in hundreds of thousands of machine-readable files (MRFs). To manage the data size for this study, we focused on UnitedHealthcare and Aetna— two national insurers that have market shares in most healthcare markets across the nation. We downloaded the payers’ raw MFRs in November 2023 and extracted raw rate files for PPO plans— the most common type of plan among the working population with employer coverage (1). To identify HOPD providers, we extracted 100% of Medicare claims to obtain a mapping of national provider identifiers (NPIs) billing for hospital and ASC services, and then linked the NPIs with the commercial rates data. Next, we constructed a preliminary study sample that includes negotiated facility prices (that is, institutional rates) for 17 ambulatory care procedures commonly performed in HOPDs, based on Healthcare Common Procedure Coding System (HCPCS) codes (see Appendix II, Figure 1).

To account for price variations due to geographic differences, we estimated the Medicare relative rates by taking the ratio between the commercial contract rate and what Medicare FFS would have paid to the same provider for the same service. We first linked the commercial rates data with National Plan and Provider Enumeration System (NPPES) data via NPI to identify the providers’ practice location (2). We then used Medicare data files on 2023 OPPS payment schedule to estimate provider-specific Medicare payment rates based on the hospital CMS Certification Number (CCN), facility location, and billing code (3, 4).

The raw data published by health plans include a large number of redundant prices due to a number of issues. For example, the same provider organization may have multiple same or different rates for multiple NPIs, places of service, and PPO plans for different employers and markets. Such data redundancy not only increases the size of the data but also poses the risk of skewing the analysis results because certain providers or services may have artificially inflated number of observations. To address this issue, we identified provider organization as the combination of TIN and ZIP code, treating all NPIs under the same TIN located in the same ZIP code are considered as one provider. We then aggregated the data by taking the median value for a given provider organization across its associated NPIs and health plans. Finally, we dropped the top and bottom 1 percent of observations based on the distribution of Medicare relative rates. After removing duplicates and outlier rates, our final study sample includes 42,145 prices on 17 billing codes for 1,932 HOPDs.

# Appendix II. Supplemental Tables and Figures

**Table 1. Summary of Medicare relative rates across providers and payers**

|  | **Observations** | **Mean** | **Standard Deviation** | **Median** |
| --- | --- | --- | --- | --- |
| **By Payer:** |  |  |  |  |
| UnitedHealthcare | 27,017 | 245% | 127% | 227% |
| Aetna | 15,128 | 243% | 151% | 209% |
| **Total** | **42,145** | **244%** | **136%** | **222%** |

HOPD = Hospital outpatient department; ASC = Ambulatory surgical center.

**Figure 1. Average Medicare relative rates by billing code**

| **HCPCS Code** | **Procedure Name** |
| --- | --- |
| 42820 | Under Excision and Destruction Procedures on the Pharynx, Adenoids, and Tonsils |
| 47562 | Under Laparoscopic Procedures on the Biliary Tract |
| 19120 | Under Ablation, Exploration and Excision Procedures |
| 49505 | Under Hernia Open Procedures |
| 29881 | Under Endoscopy/Arthroscopy Procedures on the Musculoskeletal System |
| 66984 | Under Intraocular Lens Procedures |
| 93452 | Under Cardiac Catheterization and Associated Procedures |
| 64721 | Under Neuroplasty (Exploration, Neurolysis or Nerve Decompression) Procedures on the Extracranial Nerves, Peripheral Nerves, and Autonomic Nervous System |
| 62322 | Under Injection, Drainage, or Aspiration Procedures on the Spine and Spinal Cord |
| 64483 | Under Introduction/Injection of Anesthetic Agent (Nerve Block), Diagnostic or Therapeutic Procedures on the Somatic Nerves |
| 69436 | Under Incision Procedures on the Middle Ear |
| 45385 | Under Endoscopy Procedures on the Rectum |
| 45380 | Under Endoscopy Procedures on the Rectum |
| 43235 | Under Esophagogastroduodenoscopy Procedures |
| 45378 | Under Endoscopy Procedures on the Rectum |
| 43239 | Under Esophagogastroduodenoscopy Procedures |
| 66821 | Under Incision Procedures on the Lens of the Eye |

**Table 2. Association between market concentrations and Medicare relative rates, without interactions**

|  | **Coefficient** | **Standard Error** | ***p-*value** |
| --- | --- | --- | --- |
| **HOPD Prices** (N = 41,264) |  |  |  |
| Hospital-system HHI |  |  |  |
| 2nd Tertile | 10.8%*** | 1.5% | 0.000 |
| 3rd Tertile | 26.2%*** | 1.9% | 0.000 |
| Insurer concentration |  |  |  |
| 2nd Tertile | -5.7%** | 2.1% | 0.014 |
| 3rd Tertile | -37.7%*** | 5.2% | 0.000 |

Notes: Regressions control for payer indicator, disproportionate share hospital status and major teaching hospital status, MSA-level population size and median personal income, and billing code fixed effects. Standard errors are clustered by billing codes. *p<0.10 **p<0.05 ***p<0.01

HOPD = Hospital outpatient department.

**Table 3. Association between market concentrations and Medicare relative rates, with interactions**

|  | **Coefficient** | **Standard Error** | ***p-*value** |
| --- | --- | --- | --- |
| **HOPD Prices**  (N = 41,264) |  |  |  |
| Insurer HHI 2nd Tertile | -21.8%*** | 4.2% | 0.000 |
| Insurer HHI 3rd Tertile | -60.0%*** | 5.8% | 0.000 |
| Hospital-system HHI | -7.7% | 7.2% | 0.304 |
| Insurer HHI 2nd Tertile X Hospital-system HHI | 58.3%*** | 9.9% | 0.000 |
| Insurer HHI 3rd Tertile X Hospital-system HHI | 75.3%*** | 5.7% | 0.000 |

Notes: Regressions control for payer indicator, disproportionate share hospital status and major teaching hospital status, MSA-level population size and median personal income, and billing code fixed effects. Standard errors are clustered by billing codes. *p<0.10 **p<0.05 ***p<0.01

HOPD = Hospital outpatient department.

**Table 4. Sensitivity analyses with alternative independent variables and sample inclusion criteria**

|  | **Use health system dominance instead of hospital-system HHI measure** | | | **Excluding the five largest MSAs** | | | **Including four MSAs with low system dominance with high hospital-system HHI** | | |
| --- | --- | --- | --- | --- | --- | --- | --- | --- | --- |
|  | **Coefficient** | **Standard Error** | ***p-*value** | **Coefficient** | **Standard Error** | ***p-*value** | **Coefficient** | **Standard Error** | ***p-*value** |
| **HOPDs** |  |  |  |  |  |  |  |  |  |
| Insurer HHI 2nd Tertile | -14.3%*** | 4.3% | 0.004 | -15.6%*** | 5.1% | 0.007 | -21.8%*** | 4.2% | 0.000 |
| Insurer HHI 3rd Tertile | -65.9%*** | 5.5% | 0.000 | -36.5%*** | 6.5% | 0.000 | -59.7%*** | 5.7% | 0.000 |
| Hospital-system HHI (or health system dominance) | 15.6%** | 5.9% | 0.018 | 6.5% | 7.7% | 0.409 | -7.6% | 7.2% | 0.306 |
| Insurer HHI 2nd Tertile X Hospital-system HHI (or Health system dominance) | 30.8%*** | 8.5% | 0.002 | 44.6%*** | 10.9% | 0.001 | 58.3%*** | 9.9% | 0.000 |
| Insurer HHI 3rd Tertile X Hospital-system HHI (or Health system dominance) | 80.1%*** | 4.9% | 0.000 | 30.5%*** | 7.1% | 0.001 | 73.7%*** | 5.7% | 0.000 |

Notes: Regressions control for payer indicator, disproportionate share hospital status and major teaching hospital status, MSA-level population size and median personal income, and billing code fixed effects. Standard errors are clustered by billing codes. *p<0.10 **p<0.05 ***p<0.01

HOPD = Hospital outpatient department.

**References**

1.Kaiser Family Foundation 2023 Employer Health Benefits Survey 2023 [Available from: <https://www.kff.org/report-section/ehbs-2023-section-1-cost-of-health-insurance/>.

2.Centers for Medicare & Medicaid Services NNPES NPI Files [Available from: <https://download.cms.gov/nppes/NPI_Files.html>.

3.Centers for Medicare & Medicaid Services Addendum A and Addendum B Updates [Available from: <https://www.cms.gov/medicare/payment/prospective-payment-systems/hospital-outpatient/addendum-a-b-updates>

4.Centers for Medicare & Medicaid Services ASC Payment Rates - Addenda [Available from: <https://www.cms.gov/medicare/payment/prospective-payment-systems/ambulatory-surgical-center-asc/asc-payment-rates-addenda>.
